# Advances in home-based respiratory muscle training for improving physical function in older adults with long COVID

**DOI:** 10.3389/fphys.2025.1662537

**Published:** 2025-09-09

**Authors:** Xu Guo, Xuemei Li

**Affiliations:** ^1^ School of Physical Education, Liaocheng University, Liaocheng, China; ^2^ College of Sports Medicine and Rehabilitation, Beijing Sport University, Beijing, China

**Keywords:** telerehabilitation, home-based respiratory muscle training, physical function, older adults, long covid

## Abstract

Long COVID imposes a substantial burden on older adults, manifesting as respiratory muscle dysfunction that severely compromises physical function. This narrative review synthesizes current evidence on home-based respiratory muscle training (RMT)—a non-pharmacological intervention targeting this impairment in older patients with long COVID—while critically evaluating its physiological mechanisms, therapeutic efficacy, implementation feasibility, and persistent challenges. Respiratory muscle dysfunction, caused by multifaceted neurophysiological and structural impairments, is a core mechanism of exertional dyspnea and fatigue in older adults, further aggravated by age-related decline. RMT mitigates these effects through improvements in respiratory strength, endurance, ventilatory efficiency, metaboreflex and autonomic regulation, and psychological wellbeing. Home-based RMT demonstrates non-inferior efficacy to conventional programs while providing critical accessibility for mobility-limited older adults. Nevertheless, implementation barriers include challenges in individualizing geriatric-adapted exercise prescriptions, technological access limitations, variable adherence, insufficient clinician training in remote assessment, and regulatory/policy gaps in telerehabilitation frameworks. Despite these challenges, home-based RMT represents a promising strategy for managing debilitating respiratory sequelae in this vulnerable population. This review consolidates RMT’s physiological rationale and clinical evidence, underscores its integration potential within collaborative care models, and outlines key translational priorities—including hybrid delivery systems and refined geriatric-specific protocols—to accelerate clinical adoption.

## 1 Introduction

The COVID-19 pandemic has inflicted severe global public health consequences, claiming millions of lives worldwide. Beyond exacerbating pre-existing comorbidities, the COVID-19 induces particularly significant damage to the respiratory system. This pathology can manifest as diffuse alveolar damage, frequently accompanied by microangiopathy, hyaline membrane formation, interstitial fibrosis, and pulmonary collapse (You et al., 2020; Chen et al., 2022). Consequent sequelae primarily include pulmonary dysfunction (Maldaner et al., 2021), encompassing restrictive ventilatory defects, impaired diffusion capacity, persistent pulmonary function abnormalities, and respiratory muscle dysfunction (Plaza and Sevilla, 2022). The latter is characterized by diminished respiratory muscle strength (Çelik et al., 2022) and dyspnea, collectively impairing activities of daily living and reducing quality of life among COVID-19 survivors. Furthermore, older adults with comorbidities (e.g., hypertension, diabetes) face substantially elevated risks of severe disease, frequently necessitating intensive care unit (ICU) admission (Centeno-Cortez et al., 2022). Critical illness and associated life-sustaining interventions may induce secondary injuries. These include ventilator-associated lung injury, drug-induced myopathy, and ICU-acquired weakness. Functional consequences encompass musculoskeletal pain, reduced functional capacity, depression, anxiety, and diminished quality of life. Concurrently, the inherent age-related decline in respiratory function observed in older populations (Rodrigues et al., 2021) synergistically exacerbates these impairments. Advancing age correlates with reduced thoracic elasticity and compliance, precipitating significant declines in respiratory muscle strength and pulmonary function. Additionally, older adults exhibit increased work of breathing and other manifestations of compromised respiratory efficiency.

Although medical interventions have rendered the acute symptoms of COVID-19 increasingly manageable, the long-term sequelae of the infection pose the principal threat to public health. Studies indicate (Nalbandian et al., 2021; Martínez-Pozas et al., 2024) that individuals with post-COVID-19 conditions may develop over 200 distinct symptoms during the clinical course. Among patients experiencing COVID-19 lasting >6 months, approximately 14 symptoms persist on average. Commonly reported manifestations include fatigue/asthenia, dyspnea, arthralgia/myalgia, depression, anxiety, memory impairment, impaired concentration, and insomnia (Calvache-Mateo et al., 2024). Consequently, the WHO (World Health Organization, 2025) formally defined the clinical entity “Post COVID-19 condition” (long COVID) in 2022. This condition is characterized by new-onset or persistent symptoms commencing 3 months after the initial COVID-19 infection, lasting ≥2 months, and not attributable to alternative diagnoses. Core symptoms encompass fatigue, dyspnea, musculoskeletal pain, and sleep disturbances. These symptoms significantly impair patients’ capacity to perform activities of daily living, including occupational and domestic responsibilities, and restrict social participation. The latest WHO epidemiological report (2025) (World Health Organization, 2025) estimates an incidence of 6 cases of long COVID per 100 COVID-19 infections, establishing it as a substantial and persistent challenge to global public health.

This narrative review synthesizes current evidence regarding the role, mechanisms, efficacy, and implementation of home-based RMT as a targeted non-pharmacological intervention for mitigating respiratory muscle dysfunction and improving physical function in older adults with long COVID. It further evaluates the unique advantages and challenges associated with delivering home-based RMT to this vulnerable population and identifies priorities for future research and clinical translation.

### 1.1 Search strategy

Although this review employs a narrative methodology, methodological rigor was ensured through a structured literature search to guarantee transparency and relevance. A comprehensive search was performed across PubMed, Web of Science, the Cochrane Library, and EBSCO for studies on home-based respiratory muscle training in older adults with long COVID. The search strategy incorporated Boolean operators and key terms including: “post-COVID-19 syndrome,” “long COVID,” “respiratory muscle training,” “inspiratory muscle training,” “expiratory muscle training,” and “breathing exercise.” Publications were limited to January 2020 onward, given that long COVID emerged as a clinically significant entity following the COVID-19 pandemic onset.

Inclusion criteria comprised: (1) peer-reviewed original research; (2) participants with confirmed long COVID diagnosis, irrespective of gender, ethnicity, or setting; (3) interventions delivering home-based RMT, either isolated or combined; (4) outcome measures including respiratory muscle strength, lung function, dyspnea, or functional capacity; and (5) English-language publications. Exclusion criteria covered: studies without long COVID confirmation, non-English publications, and non-empirical articles. Following title/abstract screening of studies, 14 proceeded to full-text review and satisfied all inclusion criteria (see Table 1). This transparent methodology ensures the review’s credibility and facilitates structured critical appraisal.

**TABLE 1:** Characteristics of the included studies.

Studies design	Age	Sample size (intervention/control)	Intervention (A/B/C)	Time	Times a week	Outcomes	References
RCT	50.4 ± 12.2	26 (13/13)	IMT	12 weeks	twice daily	PeakVO_2_ ↑ Quality of life ↑	Palau et al. (2022)
RCT	24(14) */40(22) *	200 (100/100)	IMT/Aerobic exercise	31 days	5 days/week	Cardiorespiratory parameters ↑ Systolic and diastolic pressure ↓ Dyspnea and lower limbs fatigue ↓ 6-min walk test ↑ Diaphragmatic thickness↑ Maximal inspiratory pressure ↑	Sánchez-Milá et al. (2023)
RCT	45.3 ± 8.0	80 (23/20/20/17)	IMT/Resistance and endurance training	8 weeks	twice daily	VO_2max_ ↑ Lower body muscle strength ↑ Dyspnea and fatigue ↓	Jimeno-Almazán et al. (2023)
RCT	48.9 ± 8.3/45.3 ± 12.8/46.5 ± 9.6/45 ± 10.2	88 (22/22/22/22)	RMT/IMT	8 weeks	6 days/week	Quality of life ↑ Inspiratory muscle strength and endurance ↑ Lower limb muscle strength ↑ Expiratory muscle strength ↑ Peak expiratory flow ↑	Del Corral et al. (2023)
RCT	46.6 ± 12.2	148 (111/37)	IMT	8 weeks	3 times/week	Health-related quality of life ↑ Breathlessness questionnaires ↓ Chest symptoms ↓ Respiratory muscle strength and fitness ↑ Aerobic fitness ↑	(McNarry et al., 2022)
RCT	38.75 ± 15.40/42.58 ± 11.40	48 (24/24)	Breathing exercises/Strength exercises	14 days	Once daily	Visual Analog Fatigue Scale ↓ 6-min walk test ↑ Thirty-Second Sit-to-Stand Test ↑ Multidimensional Dyspnea-12 ↓ The modified Borg Scale of perceived effort ↓	Rodriguez-Blanco et al. (2023)
RCT	23.33 ± 2.71/22.58 ± 2.51	48 (24/24)	Breathing exercises/Chest expansion/Aerobic activity	6 weeks	3 times/week	6-min walk test ↑ The Short Form Health Survey-36 ↑	Alsharidah et al. (2023)
RCT	49 ± 12/50 ± 12	129 (58/71)	Breathing exercises	6 weeks	once weekly	Health related quality-of-life ↑ Visual analogue scales for breathlessness ↓	Philip et al. (2022)
RCT	48.85 ± 10.85 52.19 ± 14.84	52 (26/26)	Breathing Exercises	5 weeks	3 times/day	Lung function ↑ Dyspnea ↓ 6-min walk test ↑	Okan et al. (2022)
RCT	22.2 ± 1.67/21.4 ± 1.3	35 (18/17)	Breathing exercises/Stretching exercises	4 weeks	4 times/week	Lung function ↑ Fatigue Severity Scale ↓	Jeong et al. (2024)
RCT	53.00 ± 9.84/58.08 ± 9.44	46 (23/23)	Breathing exercise/Flexibility exercise/Strengthening exercise	12 weeks	3 times/week	Peak expiratory flow rate ↑ Peak cough flow ↑ 6-min walk test ↑ Chest expansion ↑ Exercise stress test’s metabolic equivalents ↑ VO_2 peak_ ↑	Dwiputra et al. (2024)
RCT	49 ± 9	14 (8/6)	Breathing exercises/Aerobic exercises/Strengthening exercise	8 weeks	3 times/week	Fatigue ↓ Sit-to-stand capacity ↑ Neurocognitive ↑ Autonomic ↑	Sarmento et al. (2024)
Non-RCT	21.7 ± 2.3/21.1 ± 1.6	16 (8/8)	Breathing exercises/Aerobic exercises	4 Weeks	3 times/week	Oxygen saturation ↑ Heart rate ↓ Respiratory rate ↓ 6-min walk test ↑ Sit-to-stand ↑ Perceived effort dyspnea/fatigue ↓	Esparza et al. (2024)
Non-RCT	59.39 ± 16.05	18	EMST/pursed lip breathing	12 weeks	3 times/week	Pulmonary symptoms ↓ Functional performance ↑ Progressive expiratory resistance capabilities ↑	Morgan et al. (2023)

RCT, randomized controlled trial; IMT, inspiratory muscle training; EMST, expiratory muscle strength training. *: Data expressed as median (interquartile range).

## 2 Home-based respiratory muscle training in long COVID

### 2.1 Long COVID and respiratory muscle dysfunction

Dyspnea and fatigue represent cardinal manifestations of long COVID (Gomes Dos Santos et al., 2024), with exertional dyspnea emerging as the predominant clinical feature. Compelling evidence indicates (Spiesshoefer et al., 2024) that a significant subset of long COVID patients develop persistent exertional dyspnea extending beyond 28 months, despite exhibiting normal cardiopulmonary function parameters. Notably, this phenomenon mirrors established pathophysiological patterns observed in chronic respiratory diseases, where dyspnea initiates a well-characterized vicious cycle encompassing physical deconditioning, cognitive-emotional impairment, and progressive functional decline—a paradigm conventionally termed the “disability spiral” (McNarry et al., 2022). Importantly, in long COVID patients, this maladaptive cascade appears to potentiate both respiratory and fatigue-related symptoms, ultimately resulting in substantial quality-of-life impairments. While the precise pathogenic mechanisms underlying dyspnea in long COVID remain to be fully elucidated, a growing body of evidence supports a strong correlation between persistent respiratory symptoms (notably dyspnea and fatigue) and respiratory muscle dysfunction (Veldman et al., 2023; Abo Elyazed et al., 2024). This muscular impairment is modulated by various physiological and behavioral factors, including advanced age, obesity, physical inactivity, tobacco use, and pre-existing chronic conditions.

The pathophysiology of this dysfunction appears to involve both cortical neurological and peripheral muscular components. Neurophysiological investigations (De Souza et al., 2024) have revealed significant impairment in neural respiratory drive among long COVID patients, characterized by diminished cortical activation of the diaphragm. This reduction in central neural output disrupts the normal pattern of phrenic nerve stimulation necessary for optimal diaphragmatic contraction, ultimately leading to compromised ventilatory efficiency and impaired respiratory muscle performance. From a peripheral perspective, objective measurements demonstrate substantial reductions in both maximal inspiratory and expiratory pressures, strongly suggesting generalized respiratory muscle weakness with particular involvement of diaphragmatic function. Longitudinal evaluation through ultrasonographic assessment (Xavier et al., 2024) has documented persistently abnormal diaphragm thickening fractions in these patients even after 1 year of follow-up, failing to achieve values observed in healthy controls. Moreover, comparative analyses (Veldman et al., 2023) have identified distinct temporal recovery trajectories between peripheral skeletal muscles and the diaphragm following COVID-19 infection, with diaphragmatic function exhibiting a characteristically prolonged convalescence period.

**FIGURE 1:**
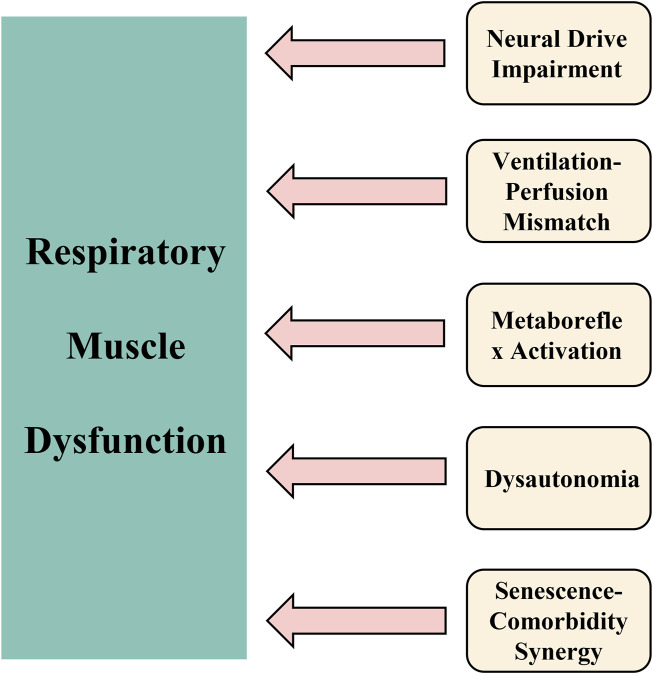
Pathophysiological framework of respiratory muscle dysfunction in older adults with long COVID. Notes: The schematic delineates five core interconnected mechanisms underlying the persistence of respiratory muscle dysfunction: (1) Upregulation of compensatory neural drive (elevated P0.1); (2) Ventilation/perfusion mismatch leading to hypoxemia and altered breathing patterns; (3) Lactate-mediated metaboreflex activation contributing to fatigue and cardiovascular adjustments; (4) Autonomic dysfunction characterized by sympathetic overactivation and impaired vagal tone; and (5) Synergistic effects of aging and comorbidities that accelerate declines in respiratory function. Collectively, these processes perpetuate a self-sustaining cycle of dyspnea, fatigue, and physical impairment.

The mechanistic basis (see Figure 1) for these observations may be explained by:

1. First, a compensatory upregulation of respiratory neural drive emerges as a key pathophysiological feature (see Figure 1, Mechanism 1). The airway occlusion pressure at 100 ms (P0.1) (Salah et al., 2022; Hennigs et al., 2022), a well-established quantitative measure of respiratory center output, shows consistently elevated values in long COVID patients. These elevated P0.1 levels reflect heightened central respiratory motor command output, serving both as a marker of increased ventilatory demand and an indirect indicator of impaired respiratory muscle function. Clinical investigations (Hennigs et al., 2022) have documented that 88% of long COVID patients demonstrate abnormally elevated P0.1 measurements, providing direct evidence of compensatory hyperactivation of the respiratory centers. This neuroadaptive response likely represents a physiological attempt to overcome either diminished respiratory muscle contractility or elevated airway resistance. Although this compensatory mechanism may preserve adequate gas exchange in the short term, chronic overactivation results in progressive respiratory muscle overload, hastens the onset of fatigue, and ultimately manifests clinically as persistent dyspnea.
2. Second, a pathophysiological ventilation/perfusion mismatch emerges as a critical consequence (see Figure 1, Mechanism 2). The impaired respiratory muscle function directly diminishes alveolar ventilation without proportional reduction in pulmonary perfusion, thereby inducing significant ventilation/perfusion inequality (Del Corral et al., 2023; Gonçalo Silva et al., 2023). This maladaptive physiological cascade evolves through distinct stages: 1) respiratory muscle weakness leads to inadequate alveolar ventilation, resulting in relative pulmonary overperfusion and subsequent hypoxemia; 2) during exertion, the compromised respiratory musculature cannot sufficiently augment tidal volume, exacerbating the hypoxemic state; 3) the progressive hypoxia potently stimulates carotid body chemoreceptors, inducing reflex tachypnea and paradoxical thoracoabdominal breathing patterns. These interconnected pathological processes collectively establish a vicious, self-reinforcing cycle of progressive respiratory dysfunction.
3. Third, a pathological metaboreflex cascade emerges (see Figure 1, Mechanism 3). Sustained high-intensity contractions of respiratory muscles (notably the diaphragm) generate significant lactate accumulation, thereby activating the diaphragmatic metaboreflex (Sze et al., 2022). This reflex arc initiates three cardinal responses: 1) tachycardia and systemic vasoconstriction, 2) inhibition of primary motor cortex output, and 3) accelerated skeletal muscle fatigue. These effects collectively establish a self-reinforcing cycle of “fatigue-metaboreflex dysregulation,” which fundamentally impairs physiological reserve. Consequently, long COVID patients manifest profound exertional dyspnea and incapacitating fatigue at minimal activity thresholds.
4. Fourth, dysautonomia constitutes a major pathophysiological component (see Figure 1, Mechanism 4). Sympathetic hyperactivation mediated through metaboreflex pathways synergistically interacts with vagal nerve impairment, establishing multifaceted autonomic dysregulation. Crucially, COVID-19 infection directly damages vagal neurofunction (Mauro et al., 2025), inducing autonomic dysfunction that manifests through three interconnected mechanisms: 1) impaired bronchodilation elevates airway resistance; 2) increased oxygen demand by compensatory respiratory muscles accelerates fatigue development; and 3) bidirectional potentiation occurs between dysautonomia and respiratory muscle dysfunction. This integrated dysregulation profoundly aggravates dyspnea in long COVID patients.
5. Fifth, pathological synergism between senescent decline and comorbidities (see Figure 1, Mechanism 5) potentiates disease progression (Reychler et al., 2016). Age-related deterioration of thoracic compliance–characterized by progressive rib cage calcification and intercostal muscle atrophy–substantially compromises chest wall mechanics, increasing respiratory work expenditure. Comorbid pathologies further exacerbate ventilatory compromise: chronic obstructive pulmonary disease drives small airway obliteration, while obesity imposes restrictive thoracic loading. These determinants converge pathologically with post-COVID structural and functional respiratory muscle injury, precipitating overt respiratory muscle dysfunction. Consequently, older adult populations demonstrate disproportionate vulnerability to debilitating long COVID symptomatology. Collectively, these mechanisms account for the heightened prevalence and refractoriness of respiratory muscle dysfunction in older adults with Long COVID, underscoring the imperative for targeted interventions.

### 2.2 Respiratory muscle training

The equations should be inserted in editable format from the equation editor. Respiratory muscle dysfunction represents a prevalent pathophysiological feature in long COVID, directly precipitating exertional dyspnea, profound fatigue exacerbation, and significantly diminished exercise tolerance during physical activity. These manifestations collectively compromise health-related quality of life and impose substantial socioeconomic burdens, while effective pharmacological management strategies remain notably limited. RMT constitutes an evidence-based non-pharmacological cornerstone intervention for respiratory muscle dysfunction, demonstrating robust efficacy in enhancing respiratory muscle strength, pulmonary function parameters, and functional exercise capacity across chronic respiratory conditions including chronic obstructive pulmonary disease, asthma, and bronchiectasis (Chen et al., 2024; Lista-Paz et al., 2023; Martín-Valero et al., 2020). Emerging evidence indicates that acute-phase COVID-19 patients receiving RMT exhibit significant improvements in diaphragmatic thickness and thoracic expansion metrics (Chen et al., 2023). Concurrently, systematic integration of RMT into long COVID rehabilitation frameworks is gaining empirical support (Jimeno-Almazán et al., 2023). Clinical investigations consistently report that RMT intervention significantly augments respiratory muscle performance, alleviates dyspnea severity, and enhances functional capacity for activities of daily living without precipitating excessive fatigue. These clinical benefits are objectively quantified through marked improvements in 6-min walk test, a functional assessment metric (Hockele et al., 2022).

The documented functional enhancements stem from RMT-induced physiological remodeling of respiratory musculature. By applying progressive external resistive loading, RMT elicits coordinated morphological and functional adaptations that collectively augment muscle strength and endurance, thereby ameliorating respiratory dysfunction. Current clinical research predominantly targets inspiratory muscle training, which imposes threshold loading during inspiration. Inspiratory muscle training directly enhances neuromuscular performance through fiber-type-specific biochemical adaptations while enabling supraphysiological training intensities without provoking clinically significant lactate acidosis. Notably, emerging evidence (Reychler et al., 2016) establishes that adjunctive expiratory muscle training confers significant improvements in integrated respiratory muscle strength and dynamic expiratory capacity—outcomes essential for pulmonary functional restitution. These benefits hold particular clinical relevance for long COVID cohorts demonstrating expiratory muscle impairment, effectively optimizing ventilatory efficiency and reducing exertional symptom burden during activities of daily living.

### 2.3 Mechanisms underlying physical functional improvement via respiratory muscle training in long COVID

1. Augmentation of Respiratory Muscle Strength and Endurance: Enhanced respiratory muscle strength derives from structural remodeling within the respiratory musculature (Hockele et al., 2022), characterized by diaphragmatic type II fiber hypertrophy—a clinically significant adaptation with particular relevance to older adults with sarcopenia, who characteristically demonstrate preferential atrophy of type II fibers (Nilwik et al., 2013)—and increased diaphragmatic thickness (Pietranis et al., 2024). Concurrently, refined neuromuscular coordination (Rodrigues et al., 2021) potentiates force production through three neurophysiological adaptations: 1) elevated motor unit recruitment thresholds, 2) increased peak firing frequencies, and 3) significantly attenuated co-activation of antagonistic muscle groups. Furthermore, RMT enhances endurance capacity by elevating the critical threshold for task failure and suppressing the respiratory metaboreflex (Morgan et al., 2023), thereby mitigating sympathetically-driven autonomic dysregulation and attenuating exertional dyspnea.
2. Optimization of Pulmonary Mechanics and Ventilatory Efficiency: RMT elicits clinically significant positive expiratory pressure (Jeong et al., 2024), serving dual physiological functions: 1) preventing dynamic airway collapse during expiration, and 2) maintaining optimal small airway patency. Mechanistically, RMT enhances pulmonary function through three coordinated effects: 1) improved ventilatory efficiency, 2) establishment of physiologically adaptive breathing patterns, and 3) substantial reduction in the oxygen cost of breathing (Palau et al. 2022). These adaptations collectively attenuate exertional dyspnea by decreasing the respiratory drive threshold.
3. Mitigation of Fatigue and Exercise Intolerance: RMT confers significant improvements in exercise capacity and autonomic cardiovascular regulation (Bostanci et al., 2019) through three synergistic physiological adaptations: 1) reduction in respiratory rate, 2) elevation of resting tidal volume, and 3) remodeling of inspiratory muscle fiber architecture. These adaptations operate via two distinct but complementary neurophysiological pathways: 1) RMT suppresses pathological metaboreflex activation in high-oxygen-demand respiratory muscles (notably the diaphragm), directly enhancing acute exercise performance. 2) The characteristic diaphragmatic breathing pattern elicited by RMT enhances arterial baroreceptor sensitivity, thereby restoring physiological sympathovagal balance. Of particular clinical relevance, RMT consistently improves first-minute heart rate recovery (HRR1) - a validated measure of autonomic recovery - across healthy populations and diverse clinical cohorts (Bostanci et al., 2019). HRR1, mediated primarily by vagal reactivation through baroreceptor and muscle chemoreflex pathways, represents a robust prognostic indicator. Delayed HRR1 portends adverse clinical outcomes in multiple populations, including healthy adults, cardiovascular patients, systemic disease cases, and long COVID patients, rendering its enhancement through RMT therapeutically meaningful.
4. Psychological Symptom Amelioration and Anxiety Reduction: RMT has been clinically demonstrated to significantly ameliorate dyspnea and fatigue symptoms while enhancing physical functional capacity in long COVID patients, collectively contributing to improved psychological wellbeing. The intervention’s psychophysiological mechanism involves coordinated strengthening of the diaphragm and deep core musculature, which generates elevated positive end-expiratory pressure and improves ventilation-perfusion matching. These cardiopulmonary adaptations mediate significant reductions in psychophysiological stress markers and produce measurable attenuation of anxiety-related symptomatology. Crucially, RMT represents a unique biopsychosocial intervention that simultaneously addresses both somatic pathophysiology and neuropsychological distress (Liu et al., 2020), culminating in statistically significant improvements in health-related quality of life metrics for long COVID patients (see Table 2).

**TABLE 2:** Multimodal physiological adaptations induced by home-based RMT.

Therapeutic target	Physiological adaptations	Objective metrics	Clinical outcomes
Strength and Endurance	Diaphragmatic type II fiber hypertrophy ↑	MIP/MEP ↑, Diaphragmatic thickness ↑	Exertional dyspnea ↓
Ventilatory efficiency	Dynamic airway collapse ↓	O_2_Cost ↓	Pulmonary function ↑
Fatigue and Exercise intolerance	Vagal reactivation ↑, Metaboreflex activation ↓	HRR1 ↑	Fatigue ↓
Psychological	Psychophysiological stress markers ↓	Anxiety ↓, Depression ↓	Mental health ↑

MIP, maximal inspiratory pressure; MEP, maximal expiratory pressure; HRR1, first-minute heart rate recovery.

### 2.4 Evidence and implementation of home-based respiratory muscle training in long COVID

In 2019, the Physiotherapy proposed an initiative to develop global standards for digital physical therapy practices and regulations (Physiopedia, 2019). This framework encompassed tele-education, telemedicine, remote monitoring, tele-assistance, and mobile health, aiming to enhance service delivery by improving access to care and information while optimizing healthcare resource management. Rapid technological advancements since then (Sarmento et al., 2024), including improved communication platforms and portable devices, have facilitated social interaction and enabled virtual programs. Wearable technology further supports patient monitoring, ensuring the safe implementation of telerehabilitation (Wootton et al., 2020). This model, widely adopted to overcome temporal and spatial constraints in treating older adult patients with cardiovascular or musculoskeletal disorders, enhances the accessibility and convenience of rehabilitation therapy compared to traditional in-person sessions. Critically, telerehabilitation demonstrates comparable efficacy to conventional rehabilitation approaches (Rodrigues et al., 2021; Dai et al., 2024). Consequently, it has emerged as an effective option for self-management rehabilitation in these patient populations. Telerehabilitation also facilitates patient self-monitoring and health education, simultaneously alleviating burdens on healthcare systems and yielding positive economic outcomes.

Furthermore, the COVID-19 pandemic has profoundly highlighted the critical importance of telerehabilitation. Research (Fisk et al., 2020) indicates persistently low global accessibility to pulmonary rehabilitation services, a situation exacerbated by the surge in demand from long COVID patients requiring respiratory rehabilitation. Concurrently, traditional hospital-based rehabilitation reliant on in-person sessions reduces participation rates among target populations and imposes additional temporal and resource burdens on overstrained healthcare systems. Consequently, telerehabilitation targeting respiratory dysfunction—particularly home-based RMT—has emerged as a vital home-based strategy, uniquely suited to the needs of long COVID patients. Existing evidence supports the efficacy of home-based RMT (Satar et al., 2023), demonstrating that its application in patients with chronic respiratory diseases enhances daily activity capacity, improves respiratory function, and boosts mental wellbeing, thereby improving quality of life. Critically, the effectiveness of home-based RMT shows no significant difference compared to conventional in-person rehabilitation (Dai et al., 2024). Furthermore, studies confirm that RMT significantly improves key functional outcomes—including pulmonary function, fatigue levels, and dyspnea—in both COVID-19 patients during the post-acute recovery phase and in those with long COVID (Chen et al., 2023). The value of RMT has been demonstrated throughout the COVID-19 pandemic. First, during peak transmission and strict containment phases, telerehabilitation served as the primary modality for patients to safely access essential rehabilitation services. Research shows that home-based RMT significantly enhanced recovery outcomes in convalescent patients with acute COVID-19 (Chen et al., 2023). Second, and more critically, home-based RMT continues to offer a vital therapeutic approach for improving physical health and quality of life in older adults with long COVID and comorbidities such as cardiovascular or musculoskeletal disorders beyond the acute pandemic phase (Fisk et al., 2020; Vieira et al., 2022).

While the optimal exercise parameters for RMT in long COVID patients remain to be fully established, its therapeutic potential remains undisputed. Robust clinical evidence confirms that diverse RMT regimens consistently yield significant improvements in patient functional status (Maldaner et al., 2021; De Souza et al., 2024; Del Corral et al., 2023; Gonçalo Silva et al., 2023; Jeong et al., 2024). This compelling evidence base highlights the critical importance of RMT—as an integral component of multidisciplinary long COVID rehabilitation programs, particularly given older patients’ heightened accessibility needs due to mobility limitations and transportation barriers.

### 2.5 Limitations of home-based respiratory muscle training in long COVID

**FIGURE 2:**
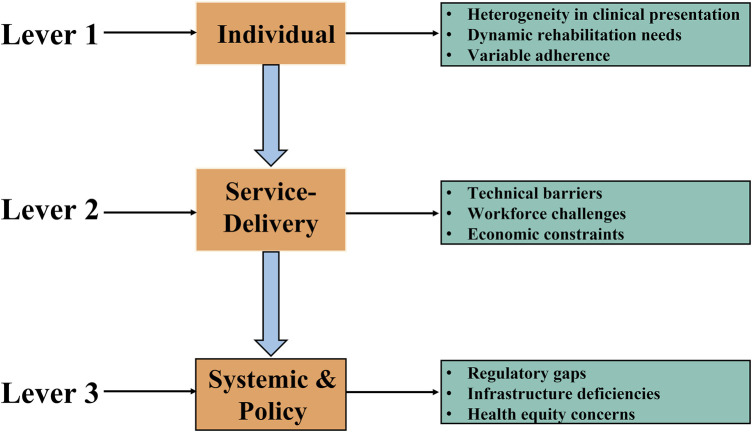
Limitations of home-based RMT in long COVID. Notes: Key challenges include the determination and dynamic adjustment of individualized exercise prescriptions, which are complicated by high clinical heterogeneity and evolving patient needs. Implementation is further complicated by fragmented data integration platforms, insufficient therapist training in remote care, and patient-related barriers such as limited digital access, low technological literacy, and suboptimal adherence. At the system level, regulatory gaps in tele-rehabilitation governance, along with technical limitations in platform reliability and interoperability, also impede broad implementation.

Although home-based RMT has demonstrated clinically meaningful benefits in ameliorating long COVID symptoms, several barriers impede its broad-scale clinical adoption (see Figure 2).

1. The determination and optimization of individualized exercise prescriptions present significant challenges (see Figure 2, Upper Panel). Firstly, optimal intervention timing and patient stratification are crucial due to the highly heterogeneous clinical presentation and varying symptom severity among long COVID patients. Research (Palacios-Ceña et al., 2024) recommends screening respiratory muscle function to identify impairment or dysfunction, enabling the prompt initiation of RMT upon confirmation. This timely intervention facilitates optimization of benefits for respiratory muscle function and associated symptom alleviation. Concurrently, stratifying patients by symptom severity is essential for identifying subgroups most likely to benefit and for guiding the prescription of appropriate training intensity. Secondly, dynamic protocol adjustment is indispensable. Significant inter-individual variability in symptoms and functional status necessitates regular, comprehensive assessments encompassing respiratory function, muscle strength, activities of daily living, and other relevant domains to ensure RMT safety and efficacy. Clinicians must dynamically adjust the home-based RMT protocol—including parameters such as intensity, frequency, and duration—based on evaluation results to address evolving patient needs throughout different rehabilitation phases.
2. The implementation of home-based RMT faces multifaceted obstacles (see Figure 2, Middle Panel). From the service provider perspective, effective data integration platforms are urgently required to provide physical therapists with ready access to essential patient health data, enabling precise prescription and optimization of training regimens. Furthermore, physical therapists require specialized training to develop proficiency in remote assessment and clinical decision-making, including the ability to judiciously determine when adjustment of training intensity is indicated or when training should be discontinued for safety reasons. Deficiencies in policy and management frameworks pose additional significant barriers beyond technical and personnel issues, including inadequate data privacy and security safeguards, as well as ambiguous reimbursement policies for telerehabilitation services, which hinder effective deployment. Numerous countries have established reimbursement mechanisms for telehealth services, including Medicare and Medicaid in the United States, federal-level public reimbursement programs in Germany, Australia, Sweden, Belgium, the United Kingdom, and Switzerland, as well as provincial-level public reimbursement for telehealth in Ontario, Canada (Huang-Ku et al., 2025). Building upon this precedent, it is imperative to elaborate more detailed reimbursement frameworks for telerehabilitation to facilitate its equitable and sustainable implementation. From the patient perspective, limited access to necessary internet-enabled devices or poor connectivity presents a practical barrier, directly impeding participation. Personal factors such as age, cognitive function, and educational level may also impact patients’ ability to utilize technological platforms and comprehend training instructions, particularly affecting octogenarians, those with low digital literacy, or older adults living alone. Variable patient acceptance of the home-based RMT model and willingness to adhere long-term further complicate widespread implementation (Semphuet et al., 2024).
3. At the systems and policy level, challenges primarily involve regulatory frameworks and technical infrastructure (see Figure 2, Lower Panel). A significant regulatory gap is the absence of detailed government-level regulations and operational guidelines specifically governing telerehabilitation services, including RMT (Palacios-Ceña et al., 2024). This absence creates ambiguity regarding the rights, responsibilities, and accountabilities of service providers, patients, and payers. Although countries such as the United States have enacted comprehensive legislation in recent years to safeguard telehealth (Shinn et al., 2025)—addressing issues such as the regulatory framework, reimbursement mechanisms, standardization, and patient privacy protection—a critical gap remains: the absence of tailored regulatory frameworks specifically designed for telerehabilitation. In the context of internet-based rehabilitation services, reinforcing system security and reliability can be achieved through the implementation of authentication protocols for telerehabilitation, thereby ensuring that all accesses are password-restricted and effectively preserving the confidentiality and privacy of patient data. Additionally, widespread technical limitations concerning the reliability, compatibility, and user experience of telemedicine platforms pose substantial constraints, hindering the broader adoption of home-based RMT.

## 3 Conclusion

Home-based RMT represents a clinically valuable non-pharmacological intervention for mitigating respiratory muscle dysfunction and enhancing physical function in older adults with long COVID. Its therapeutic efficacy—manifested through improved respiratory muscle strength and endurance, optimized ventilatory efficiency, attenuated metaboreflex activation, enhanced autonomic regulation, and alleviated psychological symptoms—confers significant clinical utility through disrupting the disabling cycle of exertional dyspnea and fatigue. Critically, telerehabilitation-based delivery addresses critical accessibility barriers for this vulnerable population, demonstrating non-inferiority to conventional programs while accommodating mobility restrictions and reducing healthcare system burdens. Although home-based RMT shows promise in improving physical function among older adults with long COVID, several important issues remain to be addressed.

Firstly, the limited number of randomized controlled trials involving older adults with long COVID constitutes a significant limitation in the existing literature. Future research efforts should prioritize two strategic directions: first, to conduct large-scale longitudinal studies with extended follow-up periods that encompass diverse subtypes of older long COVID patients, thereby improving the evidence base regarding the efficacy of home-based RMT; and second, to develop personalized training protocols specifically designed for this population. The development of such protocols should be guided by dose-response studies conducted in large geriatric cohorts to establish optimal exercise prescriptions. This includes delineating core training parameters, establishing safe intensity progression protocols, identifying key monitoring indicators during training, formulating strategies to manage interruptions in training, and implementing specific adaptations for patients with frailty or high fall risk. Lessons may be incorporated from applications of RMT in other respiratory conditions prevalent among older adults, such as chronic obstructive pulmonary disease and asthma. Secondly, further research priorities should include: the validation of biomarkers for personalizing RMT interventions, cost-effectiveness evaluations of hybrid delivery models, the development of standardized government-level telerehabilitation guidelines, and addressing challenges associated with poor interoperability and high heterogeneity across digital platforms.

Addressing these gaps will accelerate clinical translation of home-based RMT, ultimately enhancing functional independence and quality of life for the burgeoning population of older long COVID survivors.

## Abbreviations

RMT, respiratory muscle training; ICU, intensive care unit; P0.1, the airway occlusion pressure at 100 ms; HRR1, first-minute heart rate recovery; MIP, maximal inspiratory pressure; MEP, maximal expiratory pressure; RCT, Randomized controlled trial; IMT, Inspiratory muscle training; EMST, Expiratory muscle strength training.
